# The Role of Polybrominated Diphenyl Ethers in Thyroid Carcinogenesis: Is It a Weak Hypothesis or a Hidden Reality? From Facts to New Perspectives

**DOI:** 10.3390/ijerph15091834

**Published:** 2018-08-24

**Authors:** Francesca Gorini, Giorgio Iervasi, Alessio Coi, Letizia Pitto, Fabrizio Bianchi

**Affiliations:** Institute of Clinical Physiology, National Research Council, 56124 Pisa, Italy; iervasi@ifc.cnr.it (G.I.); acoi@ifc.cnr.it (A.C.); l.pitto@ifc.cnr.it (L.P.); fabriepi@ifc.cnr.it (F.B.)

**Keywords:** polybrominated diphenyl ethers, flame retardants, endocrine disruptors, thyroid cancer

## Abstract

In the last decades, the incidence of thyroid cancer has increased faster than that of any other malignant tumor type. The cause of thyroid cancer is likely multifactorial and a variety of both exogenous and endogenous has been identified as potential risk factors. Polybrominated diphenyl ethers (PBDEs), used since the 1970s as flame retardants, are still widespread and persistent pollutants today, although their production was definitely phased out in the western countries several years ago. Polybrominated diphenyl ethers are known endocrine disruptors, and the endocrine system is their primary target. Whereas animal studies have ascertained the ability of PBDEs to affect the normal functionality of the thyroid, evidence in humans remains inconclusive, and only a few epidemiological studies investigated the association between exposure to PBDEs and thyroid cancer. However, a number of clues suggest that a prolonged exposure to these chemicals might act a trigger of the most common malignancy of the endocrine system, whereas further studies with an advanced design are suggested.

## 1. Background

Since the 1970s, polybrominated diphenyl ethers (PBDEs) have been widely used as flame retardants in a variety of commercial and household products. Despite the ban on PBDE production in the European Union and the United States starting in 2004 with penta- and octa-brominated diphenyl ethers (BDE) and ending in 2013 with deca-BDE products, their resistance to degradation and potential to bioaccumulate into animal and human fat tissues, make PBDEs widespread and persistent contaminants. Polybrominated diphenyl ethers have been detected in air, soil, sediments, coastal and estuarine environments, sewage sludge, wildlife, fish and other marine life [1,2]. Polybrominated diphenyl ethers do not dissolve easily in water and bind strongly to soil or sediment particles, which reduces their migration in the groundwater but increases their mobility in the atmosphere [3]. Volatilization from soil surfaces is greater for lower brominated congeners that, once airborne, are more persistent in the atmosphere and can transported over long distances [2,3]. In addition, PBDEs already contained in existing electronic waste (e-waste) and non-e-waste landfills have been estimated to persist to nearly 2070 [4]. Human uptake primarily occurs through inhalation (indoor and outdoor air) and ingestion of contaminated food and dust [2,5], and the compounds may also cross the placental barrier, accumulating in the fetus [6]. House dust has shown to contain PBDE levels between one and two orders of magnitude higher than outdoor soils and air [6]. Levels of PBDEs measured in Americans are the highest in the world [7], in fact it has been estimated that PBDE body burden in adults in USA is 10–100 times higher than that of European and Japanese populations [8,9]. The large differences in PBDE exposure observed between European and USA populations may be due to the higher levels of PBDEs in house air and dust measured in North America (possibly a result of different fire safety standards) [6]. Conversely, in Europeans, the main pathway of human exposure to PBDEs, in particular the lower brominated congeners, is probably dietary intake, particularly meat, poultry, and dairy products [5,10]. Ingestion of house dust is critical for young children due to their tendency to play on the floors and their hands-to-mouth contact behavior [11]. Infants can be additionally exposed to PBDEs through placental transfer and breastfeeding [5,12]. Some studies found that comparing the median or mean concentration of PBDE in human milk, the levels in North America exceed the respective levels in Europe and Asia by one order of magnitude [13,14,15].

The majority of information regarding toxicity of PBDE and their metabolites derive from animal studies, though in recent years a growing number of investigations in humans suggest that exposure to PBDEs may represent an important public health issue. During the last three decades biomonitoring data has reported an increase of PBDE body burdens, with infants and toddlers showing three- to nine-fold higher levels than adults [5]. Moreover, various commercial mixtures and individual congeners of PBDEs are responsible of developmental and reproductive effects, neurotoxicity, immunotoxicity, liver toxicity, diabetes, thyroid toxicity and cancer in laboratory animals [5]. 

**Figure 1:**
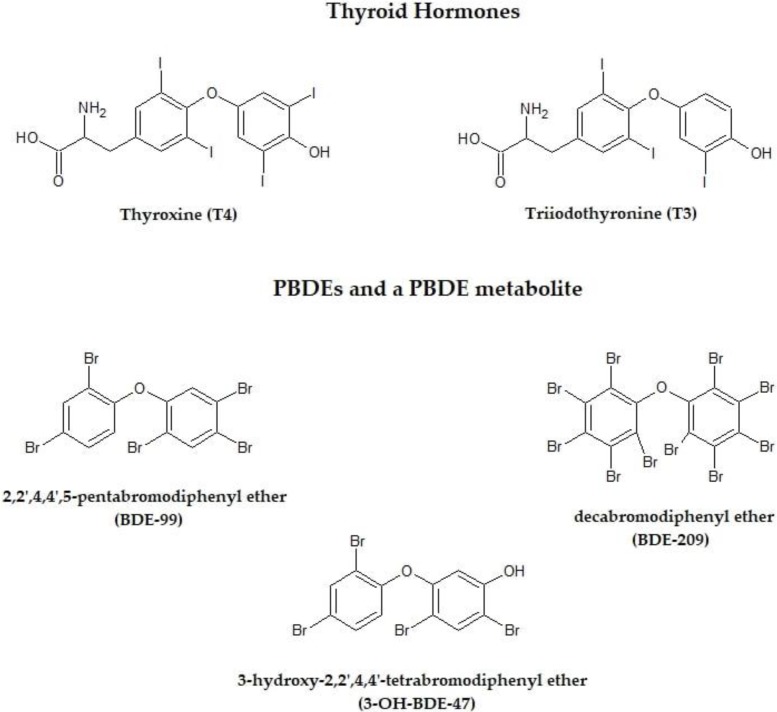
Chemical structures of thyroid hormones thyroxine and triiodothyronine and the main polybrominated diphenyl ethers (PBDEs) (modified from [16]).

Endocrine disorders represent the main effects of PBDEs, and thyroid gland is possibly the primary target because of the structure similarity of PBDEs and their breakdown products to thyroid hormones (THs) (Figure 1). Studies in rodents and fish proposed multiple ways to explain the decrease of TH circulating levels by PBDEs: (i) competitive binding to serum transporters (e.g., transthyretin (TTR) and thyroid binding globulin (TBG) replacing thyroxine (T4); (ii) reduction of proteins activity involved in TH transport; (iii) upregulation of TH metabolic enzymes [16,17]. Given the key role of THs in regulating a wide array of biological functions, the disruption of thyroid homeostasis has been suggested as a critical underlying way that could link PBDE exposure to a number of adverse outcomes in humans: goiter and other benign thyroid diseases, neurobehavioral alterations, several types of cancer including thyroid [18]. 

## 2. Thyroid Cancer: Etiology and Risk Factors Involved

Thyroid cancer has the highest prevalence of all endocrine malignancies and its incidence has continuously increased in the last three decades all over the world [19]. The rising incidence has particularly been observed in females, children and young adults [20]. In 2012, the rates of thyroid cancer incidence were highest in northern America (age-standardised rates (ASR) 6.3 in males, 20.0 in females per 100,000), and Australia/New Zealand (ASR 3.8 and 11.8 per 100,000 in males and females, respectively) [21]. In Europe, the highest incidence of thyroid cancer was recorded among men in Italy (ASR 6.7 per 100,000) and among females in Lithuania (ASR 19.3 per 100,000) [21]. From 2003 to 2007, papillary thyroid cancer (PTC) was the most prevalent histotype globally, with no significant changes for follicular, medullary, and anaplastic thyroid cancers [22,23]. In the USA, the incidence of thyroid cancer has increased faster than any other malignancy (3% per year in the period 1974–2013), and nearly all (92%) of this variation is due to the increased incidence of PTC [24], particularly the follicular variant of PTC, among all racial/ethnic groups [25,26]. In Italy, between 1998 and 2012, incidence of thyroid cancer increased of 74% in women and of 90% in men, and PTC was the most frequent histological type, showing the largest increases (+91% in women, +120% in men) [27].

The increasing incidence of thyroid cancer is mainly attributable (87%) to the detection of smaller tumors (<2 cm, and especially ≤1 cm) [28,29], and this might reflect a more frequent use of sensitive diagnostic procedures. Though “overdiagnosis” cannot be excluded, the increased incidence across all tumor sizes may suggest that increased diagnostic intensity could not be the exclusive explanation, and other factors, such as environment and lifestyle changes, likely contribute to a true increase [23,29]. Moreover, the new modes of screening and earlier diagnosis did not prevent that incidence-based mortality for PTC increased 1.1% annually in the period 1994–2013 [30] In particular, in USA thyroid cancer incidence-based mortality from 1994 to 2013 was 2.9% per year for those who were diagnosed with advanced-stage PTC [30]. 

The cause of thyroid cancer is likely multifactorial. In addition to well-recognized risk factors including age, gender, exposure to radiation, history of benign thyroid disease, and a family history of thyroid cancer [19], other endogenous (i.e., thyroid-stimulating hormone (TSH) levels, autoimmune thyroiditis, obesity, insulin resistance) and exogenous factors (i.e., iodine intake, diet, environmental pollutants) have been identified as determinants to promote mutagenesis of thyroid cells [23].

Thyroid disorders disproportionately impact women, with both benign and malignant thyroid tumors being 3–4 times more frequent in women than in men, and older women more affected than younger women [31,32]. Epidemiological, clinical, and experimental data support the involvement of estrogen and its receptors in the pathogenesis of proliferative thyroid diseases through a classical genomic and a nongenomic signaling pathway, and can explain the sex difference in the prevalence of thyroid nodules and thyroid cancer (reviewed in [32]).

Over the last decades, during the period of increasing incidence of thyroid cancer, there has been a similar trend of point mutations in the *BRAF* gene [25], which activates the RAS–RAF–MEK–MAP kinase pathway [33]. *BRAF* mutations are likely associated to high dietary iodine intake and other environmental exposures in chemical carcinogenesis [25]. In particular, *BRAF*^V600E^ that results in an amino acid substitution at position 600 in *BRAF*, from a valine (V) to a glutamic acid (E), is the most frequent genetic aberration among the tumors of classical PTC [25,34]. *BRAF*^V600E^ mutation occurs, on average, in 45% of PTC cases and was significantly associated to aggressiveness and recurrence of tumors and increased cancer-related mortality [33,35]. An Italian study reported that the incidence of thyroid cancer, and specifically of PTC, was more than doubled in residents from the volcanic area of Mount Etna (Catania province) than from the rest of Sicily [36]. In addition, among classical PTCs diagnosed in residents from Catania province, 52% was positive for the *BRAF^V600E^* gene mutation, suggesting a possible link between *BRAF^V600E^* mutation and environmental carcinogens [33,36]. Mount Etna aquifers contained a variety of metallic elements such as vanadium and ^222^radon exceeding the maximum admissible concentrations in drinking water [36]. Metals can behave both as endocrine disruptors and as carcinogens [37]. Vanadium, in particular, is classified as a possible human carcinogen (group 2B) by the International Agency for Research on Cancer [38] while for ^222^radon, which is recognized as the second most common cause of lung cancer after smoking [39], the effects on the thyroid are not known. 

Among trace elements, cadmium (Cd) is classified as a group 1 human carcinogen and is toxic to a number of tissues [40]. Besides the accumulation in the liver, kidneys, and muscles, Cd blood concentration correlates positively with its accumulation in the thyroid gland [41,42]. A Japanese study reported that Cd concentrations in the thyroid are three times higher in people who live in Cd polluted areas if compared to people residing in nonpolluted areas [43]. Cadmium has ascertained endocrine disrupting properties and both human and experimental studies reported association between Cd exposure and imbalances in plasma TH levels (reviewed in [41]). Cadmium can influence the production and/or secretion THs by follicular cells producing direct effects on the thyroid gland through induction of oxidative stress [44] or, alternatively, at extra gland level [41]. The role of Cd as etiological factor of thyroid cancer is not completely elucidated, though a recent study showed that tissue level Cd correlated with progression and severity of the disease in Korean women [45].

A plethora of persistent organic compounds exhibit endocrine-disrupting activities. Pesticides, chlorinated compounds and phenols in particular, show thyroid disrupting activities [46]. Animal studies reported decreased circulating levels of thyroxine (T4) following the treatment with hexachlorobenzene (HCB) [47], *p*,*p’*-dichlorodiphenyltrichloroethane (*p*,*p’*-DDT) [48], and 4-nonyl-phenol [49]. Although organochlorine pesticides have been banned in many countries, their metabolites are still detectable in the environment due to their long half-lives. In a cohort of newborns in China, prenatal exposure to HCB was negatively associated to TSH level in cord serum samples, suggesting organochlorine pesticides (OC) in maternal blood can transfer through the placenta [50]. Organochlorine pesticides can affect the thyroid system through gender-specific mechanisms that may differ among compounds. As shown in a cross-sectional study carried out in Brazil, β-hexa-chlorocyclohexane (HCH) and *p*,*p’*-DDT in men were associated to lower T4 levels in women, whereas increased TSH levels correlated with higher β-HCH in men [51].

Bisphenol A (BPA) is an endocrine disruptor widely used as a component of polycarbonate plastics and epoxy resins and recently banned from baby bottles in many countries by precautionary principle [46]. Mechanistic studies indicate the plausibility of BPA to interfere with thyroid function through multiple mechanisms including antagonistic binding to TH receptor and serum protein TTR [52,53,54]. Few epidemiological studies have examined the effect of BPA exposure on the thyroid and the results are inconsistent [55], with positive [56], inverse [57] or null associations [58] with TH levels. A recent prospective pregnancy and birth cohort study reported an inverse association between prenatal BPA exposure and TSH among female newborns, but not males [55]. No associations were found with maternal or cord serum TH concentrations [55], in contrast with a previous investigation that observed an inverse association between urinary BPA and T4 in pregnant women [59]. Bisphenol A is one of the highest-volume chemicals produced worldwide, thus human exposure is likely to be ubiquitous [52]. In particular, the fetus and infant appear the most vulnerable to BPA exposure due to the possibility that disruption of the thyroid axis could have implications for fetal and neonatal development and be related to child adverse outcomes [46,55].

Like BPA, phthalates—industrial chemicals applied in a large variety of commercial products— also exhibit thyroid-disrupting properties [60]. Despite the fact phthalates are rapidly metabolized and excreted in urine, their ubiquitous presence leads to an extensive human exposure [46]. Children are exposed to phthalates more widely and at higher levels than adults considering intake–body weight correlation [61]. Phthalates bind competitively to TTR [62], inhibit triiodothyronine (T3) uptake in cells [63], and reduce expression of TH receptor β gene [64]. The effects of urinary phthalate metabolite concentration on children varies in dependence of age and country. In a large cohort study of Danish children aged 4–9 years, phthalate metabolites were negatively associated with serum T3 levels [60]. In a sample of Swedish adults (≥20 years), urinary phthalates metabolite levels were inversely associated with serum T4 concentration but were positively associated with T3 and TSH levels among adolescents [65]. According to a recent mother-child pairs study performed in Taiwan, early life phthalate exposure was associated with decreased TH levels in young children of both genders [66]. Conversely, in Taiwanese children aged 9–10 years, phthalates appear to exert different effects on thyroid function in boys compared with girls [67].

The analysis of classical PTC patients residing in regions characterized by different contents of iodine in drinking water, showed that high iodine intake could represent a significant risk factor for the occurrence of *BRAF* mutations and the consequent development of PTC [68]. Iodine intake is known to influence the thyroid cancer histological type distribution. Whereas iodine deficiency is a well-established risk factor for the development of follicular thyroid cancer (FTC), iodine prophylaxis is associated with an increased risk of PTC, or a transition of FTC to an increased predominance of PTC [34,68,69]. In addition, an insufficient iodine intake appears to exacerbate effects of xenobiotics on TH levels in human subgroups [70], while in animal experiments iodine deficiency can lead to thyroid cancer through an increase of TSH, the major growth regulator of thyroid follicular cells [71]. Different signaling pathways triggered by TSH activate proliferation and differentiation of thyroid cells as well as thyroid transformation and tumorigenesis [72]. 

A number of studies showed a positive association between elevated serum levels of TSH and subsequent diagnosis of PTC [71,73]. The major limit of most of these investigations, however, was the cross-sectional design that did not permit to demonstrate the role of causation of TSH in thyroid cancer pathogenesis neither exclude the possibility of reverse causality or treatment effect [19]. Patients with well-differentiated thyroid cancers (papillary and follicular histotypes) take advantage of TSH suppression with l-thyroxine, resulting in decreased disease progression and cancer-related mortality [74]. A recent active surveillance study confirmed a persistent elevation of serum TSH levels associated with the progression of PTC microcarcinoma [75]. In contrast, the case-control study of Huang et al. reported an increased risk of PTC at TSH levels below the normal range among women and at TSH levels above the normal range among men, though the underlying mechanism of lower TSH levels increasing the risk of PTC is currently unclear [19]. 

In the last two decades the incidence of autoimmune thyroiditis, also known as Hashimoto’s thyroiditis (HT), has increased (3–6/100,000 per year) [76], and environmental pollution could play a role in such increase [12]. Hashimoto’s thyroiditis, which is the most common cause of acquired hypothyroidism, may coexist with thyroid cancer, particularly with PTC, but whether HT predisposes or protects from PTC, is still controversial [77]. In the Siracusa province (Sicily) the pollution associated to a large petrochemical complex was shown to influence the prevalence of HT that is almost two-fold greater in the contaminated area compared to the control area, with males more affected. In addition, regardless of the zone, HT was diagnosed in higher number in almost 50% of suspicious or malignant nodules [76]. Hashimoto’s thyroiditis may enhance the risk of PTC by both increasing serum TSH levels [78] and via the autoimmune inflammation process with the production of cytokines and chemokines, and generation of oxidative stress [23]. 

## 3. Effects of Polybrominated Diphenyl Ethers on Thyroid Gland 

### 3.1. Experimental Studies

Ten years ago, Zhang et al. postulated that exposure to PBDEs could increase the risk of thyroid cancer [79]. According to the Environmental Protection Agency, evidence of carcinogenic potential is suggested for decabromodiphenyl ether (deca-BDE or BDE-209) [10]. Decabromodiphenyl ether is widely distributed in the environment, and recent studies showed both the potential of BDE-209 to bioaccumulate in wildlife as well as in the general population [80]. Respect to lower brominated PBDE (e.g., those found in the penta-BDE technical mixture), PBDE-209 is more poorly absorbed and in rats 90% of the deca-BDE dose is excreted via feces [81]. Male and female rats treated with BDE-209 showed dose-related increased incidence of neoplastic nodules in the liver. A tendency to an increased incidence of thyroid adenomas or carcinomas (combined), though not significant, was observed in mice of both sexes but not in rats [82]. 

Multiple experimental studies in rodents exposed to lower-brominated congeners through oral administration in diet reported hypothyroxinemia [83]. Decreases in serum total thyroxine levels (TT4) were observed in adult animals following acute, subacute or chronic exposure to commercial PBDE mixtures [84,85,86,87,88] as well as individual congeners [89,90], whereas decreases in total triiodothyronine (TT3) were not always significant [86,89]. On the other hand, the effects of PBDEs on TSH are less consistent and decrease of TT4 in rodents were reported without increase of TSH [88,89]. In agreement with effects on THs in nonpregnant adults, perinatal PBDE exposure decreased TT4 levels in both dams and pups [91,92,93,94]. Disorders in T4 levels can also be the result of gestational exposure to PBDEs administered as a single dose [92]. Prenatal exposure to BDE-209 is able to produce significant decrease of serum TT3 levels in male mice offspring in low-dose and high-dose group [95]. Multigenerational studies of low-dose exposure levels [92,96], likely comparable to environmental PBDE levels in human exposure, showed endocrine disruption in rat offspring [97] (Table 1).

Polychlorinated biphenyls (PCBs), a group of lipophilic persistent that have not been produced for decades, and their hydroxylated metabolites OH-PCBs, also negatively affect thyroid function due to their structure similarity to T4 [46]. Hydroxylated PBDEs (OH-PBDEs) may be more potent than the parent compounds in THs disruption [98]. As shown in vitro studies, they have a stronger binding affinity to TTR than T4 and their parent congeners [99] and compete with binding of THs to human receptors [100,101]. In a similar way, OH-PCBs have a higher affinity for TTR than nonhydroxylated compounds, suggesting a critical role of hydroxylation in thyroid toxicity [102].

Combined data from studies in rodents and humans suggest that exposure to PCBs and PCB dioxin-like, which are known carcinogens [103], can lead to a decrease of circulating TH levels, especially affecting T4, and/or positive associations with TSH [46]. The relationship between PCB body burden and thyroid parameters is controversial in pregnant women and a consistent lack of association was also observed between studies analyzing the effects of prenatal PCB exposure on TH levels in newborn blood [104].

In rodents, TSH has a strong influence in thyroid carcinogenesis and its persistent elevation stimulates the thyroid gland leading to histopathological changes including follicular cell hyperplasia, considered as a precursor of thyroid tumors. A significantly increased incidence of hyperplasia of the thyroid was observed in male mice following low- or high-dose eca-BDE [82,95]. Diffuse hypertrophy of thyroid follicular cells was also reported both in male and female rats on postnatal day 20 following perinatal BDE-209 exposure, with statistically significant increases in the incidence and severity at 1000 mg/kg dose [94]. Conversely, at postnatal week 11, incidence and severity of follicular cell hypertrophy and other histopathological alterations were not statistically significant in either sexes [94]. The effects on THs in the offspring, however, are transitory and usually not evident after weaning when PBDE dosing ended [93]. Polybrominated diphenyl ethers interfere with the thyroid gland by inducing changes in morphology and histology also in fish [17]. For instance, juvenile fathead minnows subjected to dietary treatment with BDE-209 for 28 days exhibited increased thyroid follicular epithelial cell height and colloid depletion [105].

In the last few years, zebrafish (*Danio rerio*) has become a relevant model to study the biology of thyroid cancer since the main genes involved in thyroid patterning and organogenesis are well conserved with those of mammals [106]. Zebrafish embryos and larvae develop externally and are optically transparent, allowing a relatively simple experimental manipulation and observations during the entire embryonic development [106]. Recently, a novel model of thyroid carcinoma was developed in zebrafish [107]. The pharmacologic treatment using BRAF/MEK inhibitors suppressed the morphologic alterations and restored normal thyroid follicle structure in a transgenic zebrafish line expressing *BRAF*^V600E^ in thyrocytes, which had developed invasive carcinoma with diagnostic features of human PTC [107]. Though no studies ascertained carcinogenic effects of PBDEs in zebrafish, several recent investigations reported that acute or chronic administration of individual PBDE congeners or commercial mixtures affects thyroid metabolism of embryos and adult fishes and causes thyroid endocrine disruption also in the offspring of exposed fishes. In agreement with reports performed in rodents, decreased T4 levels were observed in zebrafish larvae and offspring upon BDE-209 [108,109], BDE-47 [17] and DE-71 parental exposure [110]. In addition, life-cycle exposure of environmental BDE-47 at low doses resulted in a range of morphological abnormalities and developmental retardation in larvae and offspring [17].

### 3.2. Human Studies

Results from experimental research suggested that the thyroid gland is a target of exposure to PBDEs, especially lower-brominated congeners. While in rats estimated half-lives of lower-brominated PBDEs ranges from 15 to 75 days [113], in humans half-lives of PBDE congeners ranges from 2 to 12 years [114], thus the same daily intake of PBDEs will result in tissue levels 50- to 70- times higher in humans than in rodents [113]. 

Currently, BDE-209 is the dominant PBDE measured in the environmental compartments [80]. The median concentration of BDE-209 in dust samples is 4.5 µg/g from houses and 4.2 µg/g from office in USA [115]. Decabromodiphenyl ether concentration measured in dust samples collected from commercial aircrafts can be up to two orders of magnitude (median of 495 µg/g) greater relative to other indoor environments [115]. For toddlers, exposure to PBDEs in dust through hand-to-mouth contact is nine times that of adults (median exposure estimates of 1380 and 154 ng/day, respectively) [116]. Moreover, daily exposure from all sources for children 1–5 years of age was estimated to be 13.3 ng/kg-bw/day, with approximately 77% attributable to dust [117].

In Chinese e-waste workers, serum BDE-209 concentration detected was 3100 ng/g lipid, the highest yet reported in humans [118]. Decabromodiphenyl ether was the dominant congener, accounting for 87% of ∑PBDEs in the serum of people resident in proximity of a PBDE production area [119]. In a cohort of a children from 12 to 36 months of age recruited in North Carolina, serum BDE-209 concentration ranged from <6 to 68 ng/g lipid [120]. 

In human populations, a number of studies evaluated the relationship between PBDE in body tissues and circulating TH levels, but the epidemiological evidence is inconclusive. Positive [121,122,123], inverse [124,125,126], and null associations [127,128] were reported between PBDE exposure and serum T4 levels both in adult men and pregnant women. Positive associations of PBDEs with free thyroxine (FT4) and TT4 are, however, opposite to those typically reported in laboratory animal models. Inconsistencies could be related to higher exposure levels and younger life stage at exposure in animals, the effects of acute versus chronic exposure, as well as congener-specific effects [121]. Prenatal exposure to PBDEs can lead to an increase of TSH concentration in umbilical cord blood in infants delivered vaginally [129]. In cord blood, both positive [130] and inverse correlations [125] were reported between the sum of serum PBDEs and BDE-99, and TT4 levels. An inverse association of PBDE exposure with TSH levels was observed in a cohort of pregnant women at around the 27th week of gestation [128], in contrast with the positive relationship found in 25 women at the end of the second trimester of gestation [124] and among male and female office workers [126] (Table 2). The comparison of these results, however, appears difficult because TT4 is increased by up to 50% in the first trimester of pregnancy due to estrogen-induced elevations of serum TBG [131]. Factors such as race/ethnicity, age and gender can also influence the effects of PBDEs on thyroid function [131], with higher PBDE levels measured in non-Hispanic blacks compared to non-Hispanic whites [122], and pregnant women and developing infants and fetuses being particularly sensitive populations to the effects of PBDEs [125]. Moreover, PBDE levels decrease with age, as reported in the general USA population in the 2003–2004 National Health and Nutrition Examination Survey (NHANES) for BDE-47 [120]. 

A recent meta-analysis analyzing nineteen human studies found that the relationship between PBDEs exposure and alterations in thyroid function fits an approximate U-shaped curve [132]. Other endocrine disrupting chemicals appear to have dose-responsive U-shaped [133] and/or inverted U-shaped curves [134]. The analysis of Zhao et al. described a negative correlation between PBDE exposure and TSH serum levels if the median levels of PBDEs were <30 ng/g lipid, a positive correlation if the median levels of PBDEs were >100 ng/g lipid, and no correlation in the range 30 ng/g–100 ng/g lipid. The relationship between PBDE exposure and TT4 serum levels followed a similar pattern [131].

## 4. Polybrominated Diphenyl Ethers and Thyroid Cancer: Epidemiological Evidence

Though some PBDEs are been considered carcinogenic compounds for over 20 years, evidence of PBDE carcinogenicity in humans are limited. Three case-control studies evaluated risk of different cancer, i.e., non-Hodgkin lymphoma, testicular cancer, and breast cancer, associated to PBDE exposure. Mothers whose sons were diagnosed with testicular cancer had increased serum levels of total PBDEs, but the authors did not exclude the maternal dietary intake could have caused the increase of PBDE concentration [136]. No significant associations were found between low-brominated PBDE levels in adipose tissues in women and breast cancer [103] nor increased risk of non-Hodgkin lymphoma with serum BDE-47 levels [137]. 

To date, only a few epidemiological studies assessed the correlation between PBDE exposure and risk of thyroid cancer. In a nested case-control study performed in the USA, no association was observed between increasing quartiles of the sum of serum PBDEs and single congeners (BDE-47, BDE-99, BDE-100, BDE-153) and risk for thyroid cancer, including PTC [138]. No significant differences were detected between cases and controls in lipid-corrected concentrations of ΣPBDEs or of the individual congeners (median concentration of ΣPBDE in serum blood was 12.8 and 19.4 ng/g lipid in cases and controls, respectively). The major limitation of this study is the lack of data on BDE-209, a congener whose carcinogenicity has been evaluated in animal models [138]. A recent investigation showed that patients with diagnosed PTC had higher levels of BDE-209 measured in dust from their home environment than age- and gender-matched controls, whereas, in accordance with the previous study, levels of two penta-BDEs (BDE-47 and BDE-153) measured in >70% of serum samples did not exhibit a significant association [139]. Decabromodiphenyl ether appeared to contribute to the onset of smaller and less aggressive tumors, and associations varied depending on the presence of *BRAF*^V600E^ mutation, thus participants with higher levels of BDE-209 in house dust were more likely to be *BRAF* (-) cases than controls [139]. 

**Figure 2:**
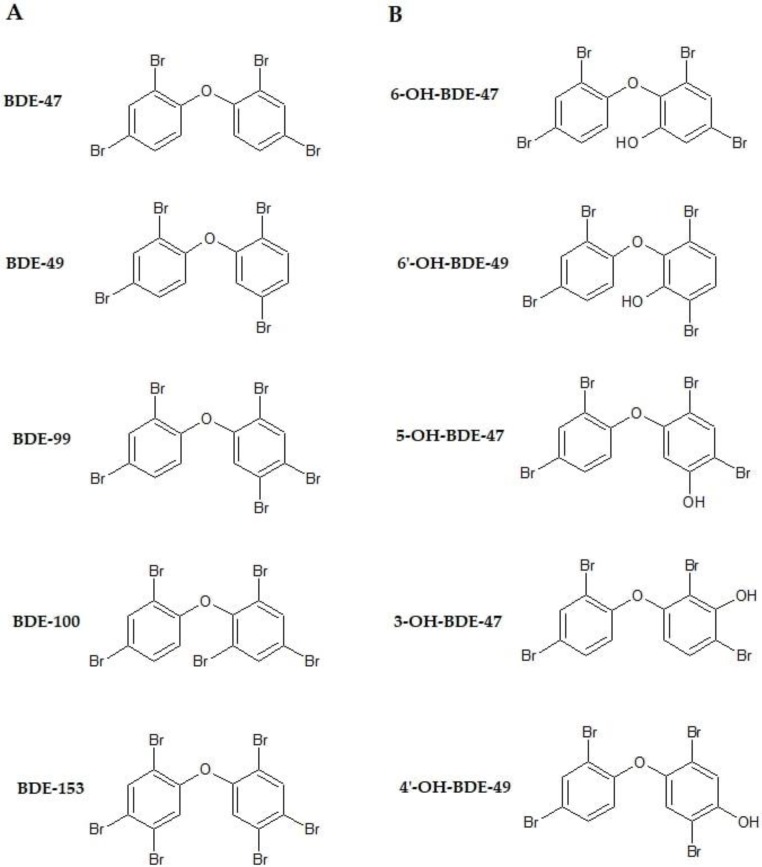
Molecular structure of (**A**) the PBDEs (BDE-47, BDE-49, BDE-99, BDE-100, BDE-153) and (**B**) the metabolites of BDE-47 (6-OH-BDE-47, 6′-OH-BDE-49, 5-OH-BDE-47, 3-OH-BDE-47, and 4′-OH-BDE-49) (modified from [141]).

Liu et al. [140] evaluated for the first time the association between serum levels of OH-PBDEs, deriving from human liver metabolism of PBDEs [124], and FT4, free triiodothyronine (FT3) and TSH levels in a cohort of 33 Chinese PTC patients. The serum concentrations of seven PBDEs and 11 OH-PBDEs were measured: all the congeners were detected in >50% of serum samples (median concentration of ΣPBDE and ΣOH-PBDEs of 4.46 and 0.06 ng/g lipid, respectively), and 6-OH-BDE-47 was the most frequently detected congener (84.8%) among all OH-PBDEs investigated. After adjusting for sociodemographic characteristics and using log transformed data, the results showed that hydroxylated metabolites of PBDEs were significantly associated with reduced FT4 and increased TSH in the thyroid cancer population. No significant relationships were observed between FT3 and any of the congeners examined, in contrast to the study of Stapleton et al. [122] who reported a significant inverse association of TT3 levels above the normal range in pregnant women with 4′-OH-BDE-49 and ΣOH-PBDEs. In another cohort of pregnant women, individual OH-PBDEs and their sum were positively associated with TSH, with the strongest association for 4′-OH-BDE-49. An inverse association, though not significant, was also reported between 6-OH-BDE-47 and TT4 (128] (Figure 2).

## 5. Discussion and Conclusions

Animal studies have ascertained that PBDEs have thyroid-disrupting properties, resulting in decreases of serum TH levels in a dose-dependent fashion and histopathological alterations. In contrast, PBDE exposure generally did not affect TSH levels. It is of importance to perform in vivo experimental studies to evaluate the health effects of ingestion of PBDE mixtures present in the environment at doses that might be relevant to human exposure. Most studies were conducted using high doses (>3 mg/kg) of PBDE commercial mixtures or single congeners, whereas in few studies PBDEs were administered to animals at low doses that can be predictive of environmental human exposure. Despite the obvious limitations of investigations performed on laboratory animals regarding both the dosage used and the frequent low statistical power, they provide some important information of an environmentally relevant class of thyroid disrupting chemicals for human and ecological risk assessment. Whereas most experimental studies showed decreased T4 levels following PBDE exposure without affecting TSH concentration [88,89], epidemiological research reported both a positive relationship between PBDEs and T4 levels [121,122,123] and the opposite, in other words a positive association of lower brominated PBDE congeners with TSH levels [124,126,129]. These discrepancies can be attributable to differences in the congeners examined and exposure levels, as well as to physiological differences between rodent models and humans. For example, hydroxylated PBDEs are able to displace T4 from the binding to TTR [142], but it is unclear whether they bind TBG, the major T4 binding protein in humans [135].

Conflicting results in epidemiological studies on thyroid alterations from PBDEs can be explained by the wide array of factors that can influence the direction of association: the study design, the method of measuring free TH levels (e.g., immunoassays, direct equilibrium dialysis), the congeners of PBDEs examined (e.g., parent compounds or hydroxylated metabolites), the different distribution of variables affecting susceptibility between study populations (e.g., age, gender, ethnicity, iodine intake, presence of HT, different genetic polymorphisms affecting PBDE metabolism, and other environmental exposures) [98]. Differences in exposure times (e.g., TH levels fluctuate throughout pregnancy), and routes of exposure is an additional critical factor that may partly explain heterogeneity of results among human studies [41]. Further investigations on environmental PBDE exposure and human thyroid function are needed and should include a more comprehensive exposure assessment with the prospective follow-up of vulnerable populations such as children in which PBDEs is of concern to their effects on neurodevelopment. The use of handwipe samples as a matrix to examining exposure to PBDEs in dust could be a valuable method especially in children, which be more exposed to contaminants in house dust from their hand-to-mouth activities [120]. Penta-BDE levels in handwipe and serum samples are correlated, but while serum concentrations of these compounds should reflect long-term exposure, handwipe samples presumably reflect a more recent exposure [143].

To date, only a few epidemiological studies have investigated the role of PBDEs as risk factors of thyroid cancer. Independently of the underlying mechanisms, increases in TSH levels is likely correlated to increased risk of thyroid cancer [23], thus we can primarily hypothesize that high and prolonged exposures to persistent chemicals such as PBDEs, via the elevation of serum TSH levels, might represent a potential additional causal factor involved in the carcinogenesis process. In addition, according to the meta-analysis of Zhao et al., the correlation between TSH circulating levels and PBDEs is strictly dependent on intensity and duration of PBDE exposure, with a positive relationship observed when PBDE serum levels were >100 ng/g lipids [132].

In the environment, both animals and humans can be exposed to a mixture of persistent compounds such as metals and organic chemicals with known or suspicious thyroid disruptors activities and/or carcinogenic effects, but almost all available toxicity data are produced from studies on single compounds. Due to the multifactorial etiology of thyroid cancer, however, additive and/or synergistic effects among different risk factors and/or different chemicals [125] must be taken into account, as well as the possibility of the further action of new, still unidentified carcinogens. In volcanic areas, there is approximately one hundred of naturally occurring elements—many of which can be toxic to humans at high doses [144]—and thyroid cancer incidence is markedly increased [145]. Thus, chronic exposures to a mixture of chemicals with carcinogenic potential can explain the increase in *BRAF* mutations associated to PTC cases observed all over the world.

Hence, epidemiological studies through advanced design with individual exposure assessment are strongly recommended, possibly using human biomonitoring data and pharmacokinetic models. The investigation of cofactors of exposure to PBDEs and other pollutants with endocrine disrupting properties today it seems a necessity more than an option of choice. At the same time, combined data from animal models that accurately define the mechanisms of action and potential direct effect at DNA level are required to clarify the exact role of PBDEs in human disease and plan preventive measures and surveillance systems. In addition, given the critical role of THs in growth and neurological development, the understanding of PBDE action on thyroid function may be of greater relevance in children to prevent adverse outcomes. In this context, in Italy, an ongoing biomonitoring epidemiological study has been recently funded in order to investigate the role of pollutants, specifically Cd, lead (Pb), and PBDEs, as factors involved in increasing risk of thyroid disorders and thyroid nodules in a child-bearing age healthy sample from Milazzo area and a control area, in Sicily region. Milazzo is a National Priority Contaminated Site (NPCS) at high risk of environmental and health crisis due to the widespread pollution principally produced by a large oil refinery. During the period 1996–2005, an excess for thyroid cancer was reported in both genders in Milazzo and other three NPCSs [146]. Primary objective of the research is the evaluation of the relationship between environmental exposure and evidence of thyroid nodules >1 cm (lesions for which guidelines from the American Thyroid Association recommends needle biopsies [147]) as biomarkers of early damage to thyroid gland. Measurements of THs and detection of Cd and Pb in blood and urine samples as well as PBDEs in blood of a subgroup of enrolled subjects, will be also provided. The peculiarity of this project is to support results from human biomonitoring with experiments carried out in Zebrafish, a model system in which acute or chronic administration of both BDE-209 and BDE-47 was reported to strongly impact thyroid metabolism of embryos and adult fishes leading to thyroid disruption also in the offspring [17,108,109]. Thus, zebrafish at fry stage of development will be exposed for 7 days to different BDE-47 doses and coexposition of BDE-47 and Pb will be further tested. One such approach including both a biomonitoring study based on the evaluation of early biomarkers of disease and in vivo experimental research would provide an integrated approach allowing to deepen the mechanisms of action of PBDEs and their role in the etiology of thyroid cancer.

## Figures and Tables

**Table 1:** Summary of studies in rodents analyzing the effects of polybrominated diphenyl ethers (PBDEs) on thyroid function.

Treatment	Species	Dose/Duration	Effects Observed	Reference
DE-71	Female C57BL/6 mice	Acute single doses: 0, 0.8, 4.0, 20, 100, 500 mg/kg by diet; 1 day Subchronic daily doses: 0, 250, 500, or 1000 mg/kg/day by diet; 14 days	Lower TT4 levels except at 100 mg/kg dose Decrease of TT4 and FT4 levels in a dose-dependent manner.	[84]
DE-71, DE-79, DE-83R	Female Long-Evans rats	0, 0.3, 1, 3, 10, 30, 60, 100, or 300 mg/kg/day by diet 14 days	Dose-dependent depletion of TT4 following exposures of DE-71 and DE-79 TT4 was decreased a maximum of 80% for DE-71 for the 300 mg/kg dose and 70% for DE-79 for 100 mg/kg dose TT3 was decreased of 30% for the 300 mg/kg dose and 25% for DE-79 for 100 mg/kg dose	[85]
DE-71	Primiparous Long-Evans rats	0, 1, 10, or 30 mg/kg/day by diet; GD6-PND22	Reduction of TT4 in dose-dependent manner in fetuses on GD20 Reduction of TT4 in GD20 and GD22 dams exposed at 30 mg/kg/day On PND 4 and PND 14 significant dose-dependent decreases of TT4 at 10 and 30 mg/kg/day doses No significant effects on TT3 in either the dams or offspring.	[91]
DE-71	Wistar rats (RIVM/Cpb:WU) of both sexes	0, 0.27, 0.82, 2.47, 7.4, 22.2, 66.7 or 200 mg/kg/day by diet 5 days	Lower TT4 levels No effects on circulating TT3	[86]
BDE-99	Wistar rats	0.06 or 0.3 mg/kg by diet GD6	Lower T4 and T3 levels in exposed dams at 0.3 mg/kg. Lower T4 levels in male and female offspring on PND 22 at 0.3 mg/kg dose. Lower FT4 levels in female pups on PND22 (0.06 mg/kg)	[92]
DE-71	Male and female Wistar rats	0, 3, 30, or 60 mg/kg/day by diet PND 23–53 and 23–28 in males; PND 22–41 and 21–26 in females	Lower TT4 levels in the 30 and 60 mg/kg dose groups following the 5-day and 21-day exposures in females. Lower TT4 levels at 3, 30, and 60 mg/kg doses in 31-day exposed males. Lower TT3 and higher TSH levels in 30 and 60 mg/kg doses in the 31-day exposed males.	[87]
DE-71	Pregnant Long-Evans rats	0, 1.7, 10.2, or 30.6 mg/kg/day by diet GD6-PND21	In dams, lower TT4 levels in the 10.2 and 30.6 mg/kg dose groups In both male and female offspring, age-dependent decrease in TT4 levels at 30.6 mg/kg/day dose No significant effects for maternal TT3 levels in dams and offspring	[111]
BDE-47	Pregnant Sprague–Dawley rats	BDE-47 (0, 1, 5, 10 mg/kg) and/or PCB153 (5 mg/kg) by diet PND10	Lower T4 levels in the 5 mg/kg BDE-47 + 5 mg/g PCB153 group compared to the 5 mg/kg BDE-47 group Lower T4 levels in the 10 mg/kg BDE-47 + 5 mg/kg PCB153 group compared to the 5 mg/kg BDE-47 group No significant alterations for T3 and TSH levels	[89]
52.1% DE-71, 0.4% DE-79, 44.2% decaBDE-209,	Adult male Sprague Dawley rats	0, 0.02, 0.2, 2, or 20 mg/kg/day by diet 70 days	Lower T4 levels in the 20 mg/kg dose group No significant effects on TSH levels	[88]
BDE-99	Male and female Sprague Dawley rat	0, 1 or 2 mg/kg/day by diet GD6-PND21	Lower FT4, T4 and T3 levels in the 2 mg/kg dose group	[90]
DE-71	Pregnant Sprague–Dawley rats	0, 0.3, 3.0 or 30 mg/kg/day by diet GD1-PND21	Lower T3 and T4 levels in dams only in the 30 mg/kg dose group. In male and female pups 3.0 and 30 mg/kg doses decreased T4 and TSH levels at PND21 In male and female pups only 30 mg/kg doses decreased T3 levels	[93]
DE-71	Male and female rats (CD^®^IGS)	0.06 mg/kg/day by diet GD1.5-PND20 (except the day of parturition)	Greater TT4 and TT3 levels in pregnant F1 offspring (GD14.5). No significant effects on TT3 and TT4 levels in the F0 mothers and pups	[96]
BDE-209	Pregnant Sprague–Dawley rats	0, 10, 100, 1000 mg/kg/day by diet PND10-PND42	Lower T3 and T4 levels in male offspring at the highest dose	[94]
BDE-209	Adult male Sprague Dawley rats	0, 100, 300, 600 mg/kg/day by diet PND10-PND42	Lower T3 levels only in the 300 and 600 mg/kg BDE209 groups. Higher TSH levels in the 300 and 600 mg/kg dose groups	[112]
BDE-209	Adult male and female CD-1 mice	0, 10, 500, or 1500 mg/kg/day by diet GD0-GD17	No significant changes in T4 levels in male offspring. Significant reduction of T3 levels 20.6% for 10 mg/kg and 20.7% for 1500 mg/kg group) in male offspring at PND71	[95]

DE-71: penta-, tetra-, hexa-, tri-BDE; DE-79: octa-, hexa-, penta-, tri-BDE; DE-83R: deca-BDE (>98%); GD: gestational day, PND: postnatal day, T4: thyroxine, TT4: total thyroxine, TT3: total tridiothyronine, T3: triiodothyronine, TT3: total triiodothyronine, TSH: thyroid-stimulating hormone, FT4: free thyroxine.

**Table 2:** Summary of human studies on association between PBDE exposure and thyroid parameters.

Study Design	Country	Study Sample	Sample Size (*N*)	Age (Years)	Main Results	Exposure Assessment Matrix/Chemical Concentration	Confounders	Reference
Prospective cohort	USA	Great Lakes anglers (non-Hispanic White)	36	29–45	No statistically significant associations among nine PBDE congeners or their sum, and either TSH or FT4	Serum blood BDE-47: median 7.9 ng/g lipid Sum PBDE: median 15 ng/g lipid LOD range: 0.002–0.028 ng/g lipid	(1), (14), physician-diagnosed goiter or thyroid condition, use of thyroid-active pharmaceuticals at the time of blood donation, having ever worked with or near plastics	[127]
Retrospective cohort	USA	Pregnant women. Blood samples collected at 27.3 ± 3.1 weeks’ gestation	270	18–45	None of the five PBDE congeners or their sum significantly associated with FT4 and TT4 concentrations. All PBDE congeners (BDE-28, 47, 99, 100, 153) significantly inversely associated with TSH (10.9–18.7% decrease in TSH for a 10-fold increase in serum concentration of individual congeners).	Serum blood Sum PBDE: GM 26.5 ng/g lipid, median 25.2 ng/g lipid LOD range: 0.2–1.6 ng/g lipid	(1), (2), (3), (4), (5), (6), (7), (8), (9) at the time of blood collection, (14), drug consumption during pregnancy, blood lead, serum PCB, organochlorine pesticide concentrations	[128]
Retrospective cohort	USA	Pregnant women. Blood samples collected at 27 3 ± 3 weeks’ weeks’ gestation (*n* = 209) and at 40 ± 2 weeks’ gestation (*n*=80). TSH levels measured in infants within 24 h after birth	289	18–45	No statistically significant associations between maternal total serum PBDE concentrations and neonatal TSH levels	Serum blood Sum PBDE: GM 28,0 ng/g lipid, median 25.4 ng/g lipid LOD range: 0.2–2.6 ng/g lipid	(1), (2), (3), (6), (7), (9), (10), (11),(12), (13) duration of residence in US, serum levels of total PCBs, HCB, DDT, and DDE	[135]
Cross-sectional	USA	Women with singleton deliveries. Umbilical cord blood collected at delivery	92	14–43	For infants born by spontaneous, vaginal, unassisted deliveries, BDE-47 significantly correlated with increased TSH levels in cord blood. PBDEs showed a negative association (not statically significant) with FT4 and TT4.	Serum blood BDE-47: mean 14.4 ng/g lipid, median 13.8 ng/g lipid Median LOD: 1.3 ng/g lipid	(1), (2), (3), (4), (8), (9), (10), (13), maternal socioeconomic status; history of STDs, hypertension, diabetes, and anemia	[129]
Prospective cohort	USA	Great Lakes fish consumers (adult males)	308	30–59	ΣPBDEs significantly and positively associated with TT4, FT4, urinary T4, rT3, and albumin-bound T4, and was negatively associated with TSH and TT3 Similar results for BDE-47, the dominant PBDE congener ΣPBDEs positively related to the percentage of T4 bound to albumin and inversely related to the percentage of T4 bound to TBG	Serum blood Sum PBDE: GM 27.7 g/g lipid, median 38 ng/g lipid LOD range: 0.025-0.15 ng/g lipid	(1), (4), (5), (14), medication use, Great Lakes sport fish meals in the past year, sport fish meals in the past year, ΣPCBs, DDE, years consuming sport fish meals, years consuming Great Lakes sport fish meals, HA1c level, levels of testosterone, SHBG, and SHBG-bound testosterone	[121]
Prospective cohort	USA	Pregnant women (80% non-Hispanic black) Blood samples collected at >34 weeks’ gestation	137	18–39	TT4 positively and significantly correlated with BDE-47, 99, 100, and ΣPBDEs FT4 positively and significantly associated with BDE-47, 153, and ΣPBDEs No significant association between TSH, TT3 or FT3 and PBDEs	Serum blood: BDE-47: GM 16.5 ng/g lipid	(1), (2), (4), (8), (9)	[122]
Cross-sectional	USA	Pregnant women. Blood samples collected prior to second trimester pregnancy termination	25	16–45	Positive significant association between ΣPBDE_5_ and TSH levels Slightly negative association between BDE-28 and FT4 Individual OH-PBDEs and their sum positively associated with TSH Relationships between OH-PBDEs and TT4 and FT4 null except for 6-OH-BDE-47 (not significant inverse association)	Serum blood: Sum PBDE: GM 85.8 ng/g lipids; BDE-47: GM 47.1 ng/g lipid	(1), (2), (8), type of health insurance	[124]
Prospective cohort	USA	Men from an infertility clinic. Blood and house dust samples	24	18–54	Positive association of PBDEs with FT4.	House dust: BDE-47: GM 577 ng/g BDE-99: GM 809 ng/g BDE-100:GM 220 ng/g LOD: 83 ng/g	(1), (14)	[123]
Prospective cohort	Canada	Pregnant women. Blood samples collected at <20 weeks’ gestation for analysis of PBDEs and THs. Maternal blood and umbilical cord blood collected at delivery for TH analyses	387	17–40	At <20 weeks’ gestation TT4 and TT3 were negatively related to BDE-47, BDE-99, and ΣPBDE. Serum TSH was not related to PBDEs. A positive relationship was observed between FT4 and PBDE-47, PBDE-99, and ΣPBDE and between FT3 and PBDE-99 and ΣPBDE At delivery, maternal TT4 decreased in relation to BDE-99. A negative association was observed between FT3 and BDE-47. No relationships were observed between TT3, TSH and PBDE congeners. In umbilical-cord blood, TT4 and FT4 levels decreased in relation to BDE-47, BDE-99, and ΣPBDE	Serum blood Sum PBDE: median 30.92 ng/g lipid BDE-47: median 21.47 ng/g lipid	(1), (4), (5), (9), (13), (14), (15), (16), blood selenium, blood mercury, medication use, familial history of hypothyroidism, and occupational and recreational exposures to chemicals	(130]
Prospective cohort	USA	26 male and 25 female adult office workers Serum samples at approximately six-month intervals from January 2010 to May 2011. Urinary samples	51	20–≥60	Significant, inverse associations between PBDEs (BDE-28, 47, 99, 100, 153) and serum TT4. Associations of PBDEs with TSH positive but small and not statistically significant. Any important associations between PBDEs and FT4 or TT3.	Serum blood Sum PBDE: Sample 1 GM 22 ng/g lipid; sample 2 GM 23 ng/g lipid; sample 3 GM 19 ng/g lipid LOD range: 0.2–0.8 ng/g lipid	(1), (14), (15), (16), sex, oral contraceptives, urinary perchlorate, urinary thiocyanate, urinary specific gravity	(131]
Prospective cohort	China	Pregnant women. Cord blood samples collected immediately post-delivery	123	≤25–≥35	BDE-99 and Σ_4_PBDEs (the sum of BDE-47, 99, 100, and 153) were associated with increased TT4 levels.	Cord blood (*n *= 106): BDE-47: GM 4.34 ng/g lipid, median 3.96 ng/g lipid BDE-99: GM 9.90 ng/g lipid, median 15.85 ng/g lipid	(1), (4), (8), (9) (10), (14)	(132]

BMI: body mass index, PBDEs: polybrominated diphenyl ethers, OH-PBDEs: hyrdroxylated polybrominated diphenyl ethers, FT4: free thyroxine, TT4: total thyroxine, T4: thyroxin, TSH: thyroid-stimulating hormone, rT3: reverse triiodothyronine, FT3: free triiodothyronine, LOD: limit of detection, GM: geometric mean, PCB: polychlorinated biphenyls, HbA1: Hemoglobin A1c; HCB: hexachlorobenezene, DDT: dichlorodiphenyl trichloroethane, DDE: dichlorodiphenyl dichloroethane, SHBG: sex hormone-binding globulin; STDs: sexually transmitted diseases, TBG: thyroid binding globulin, LOD: limit of detection, GM: geometric mean. (1): age, (2): maternal race/ethnicity, (3): maternal education, (4): smoking, (5): alcohol consumption, (6): family income, (7): country of birth, (8): parity, (9): gestational age, (10): infant sex, (11): birth weight, (12): marital status, (13): mode of delivery, (14): BMI, (15): thyroid peroxidase antibodies, (16): urinary iodine concentration.
